# Suspected case of benign familial fleck retina with functional loss

**DOI:** 10.1002/ccr3.8362

**Published:** 2023-12-21

**Authors:** Paul A. Constable, Lynne Loh, John R. Grigg

**Affiliations:** ^1^ Flinders University, College of Nursing and Health Sciences Caring Futures Institute Adelaide South Australia Australia; ^2^ Save Sight Institute, Faculty of Medicine and Health University of Sydney Sydney New South Wales Australia

**Keywords:** genetics and genomics, ophthalmology, physiology

## Abstract

**Key Clinical Message:**

Inherited retinal dystrophies typically affect vision in early childhood; however, this case highlights a late onset retinal dystrophy presenting in midlife and the need for extended visual electrophysiology testing to determine the etiology.

**Abstract:**

A 53‐year‐old female was referred for visual electrophysiology following a routine optometric eye examination in which yellow flecks were noted in both fundi and the patient had reported a recent near accident whilst driving at night. There was no reported family history of eye disease. Retinal examination identified bilateral yellow punctate and irregularly shaped lesions throughout the posterior poles sparing the macula region. Fundus autofluorescence showed coinciding hyperfluorescence with the lesions and bilateral hypofluorescent crescents superior to the macular with corresponding retinal thinning. Visual fields and color vision were normal. ISCEV standard 20 min and extended 60‐min dark adapted electroretinograms were recorded. Recovery to normal b‐wave amplitudes was noted in the DA0.01 flash but reduced a‐wave amplitudes were noted in the DA3 and DA10 flash following both dark adapted periods. Cone function was reduced but within normal limits. Genetic screening revealed a previously unreported variant of unknown significance in the gene *PLA2G5*:c.40 + 5del (rs1364254561) which is a member of the phospholipase A2 family and is associated with familial benign flecked retina.

## Graphical abstract

Fundus images illustrating discrete yellow flecks in the retina with corresponding hyperfluorescence of the retinal pigment epithelium.

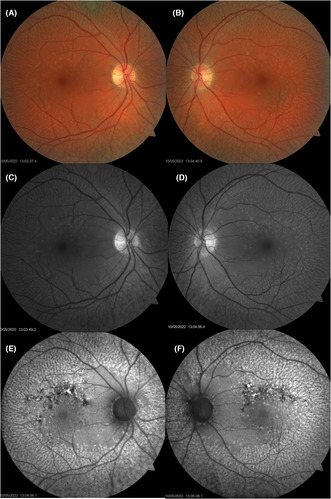

## 1 INTRODUCTION

Familial Benign Fleck Retina (FBFR) (OMIM #228980) is an autosomal recessive condition caused by biallelic mutations in the gene *PLA2G5* that encodes a family V phospholipase A2 which has multiple roles including signal transduction, phospholipid metabolism, and regulating phagocytosis.^1^, ^2^, ^3^, ^4^, ^5^, ^6^ This rare condition has been observed predominantly in children and young adults that present with yellow to white symmetrical flecks bilaterally with otherwise normal acuity, visual fields, and retinal function.^7^, ^8^

Isaacs et al.^8^ reported a case in a young female of an Australian aboriginal and Caucasian decent whose fundi had multiple yellow‐white flecks sparing the macula with only a slight delay in the left eye of the 30 Hz flicker with normal dark‐ and light‐adapted flash electroretinograms (ERGs). Audo et al.^9^ reported a case of an Indian boy aged 6 with bilateral flecks in the retina and normal electrophysiology whose parents were third cousins. Bin et al.^10^ found an association with mutation in exon 3 of *PLA2G5* (p.Gly45Cys) resulting in discrete bilateral yellow flecks in one affected triplet. Garcia et al.^11^ described a case of FBFR in a female 27‐year‐old with normal dark and light adapted ERGs and electrooculogram, although the authors noted a “selective reduced amplitude of the oscillatory potentials” but did not quantify these observations. Reduced mfERGs amplitudes and reduced sensitivity using microperimetry have been observed in FBFR but typically full‐field ERGs and electrooculograms are normal.^12^ Neriyanuri et al.^13^ reported four cases aged from 19 to 35 years with flecked retina and normal ERGs except one case who had a reduced light‐adapted ERG with two cases reporting parental consanguinity. Electrooculograms were normal but color discrimination was reduced in two cases, and all had normal visual acuity. Most recently, a presentation of FBRF was reported incidentally in a 44‐year‐old female who presented with a unilateral posterior scleritis.^14^

We report a suspected case of FBFR in a 53‐year‐old female who presented with early stages of nyctalopia, flecked retina, a family history of consanguinity in her great‐grandparents. In contrast to previous cases of FBRF, this subject had and reduced dark‐adapted a‐wave amplitudes with a variant of unknown significance in *PLA2G5*.

## 2 CASE HISTORY/CLINICAL EXAMINATION

A 53‐year‐old female of Italian ethnicity from the villages of Santa Croce del Sannio and Morcone in the Campania region. She was referred for visual electrophysiology following a routine optometric eye examination in which yellow fleck like lesions were noted in both fundi. Ophthalmic review was prompted following a near collision at night with a cyclist that “she did not see” to her right. A previous ophthalmic examination had identified retinal flecks 5 years previously. Fluorescein angiography was normal at that presentation. This presentation, in March 2022, found her refraction to be: RE +0.50/−0.25 × 177 and LE +0.75/−0.50 × 87 Add +2.25, visual acuities 6/6 OU and Goldman applanation IOPs of 13 mmHg OU. Visual fields HFA‐II 24–2 were normal with mean deviation RE −0.03 dB and LE −0.90 dB. Color vision (binocular) with the D‐15 was normal. The patient reported a marriage between cousins in her grandparent's lineage, but no family members had reported any ocular disorders, and her two children had no significant ophthalmic history.

**FIGURE 1:**
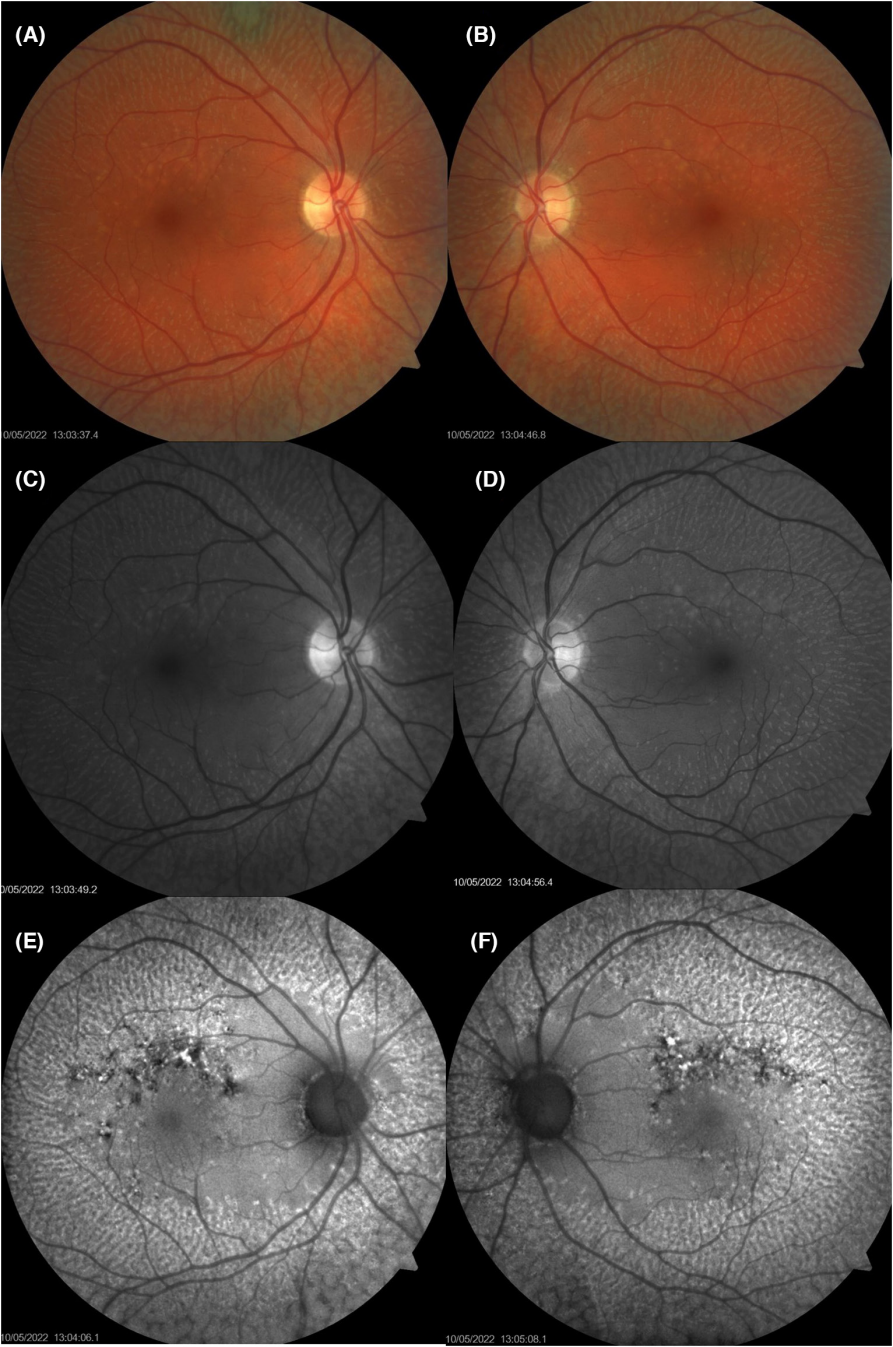
Upper rows (A, B) shows fundus photos with multiple yellow flecks and punctate lesions scattered throughout the retina but sparing the fovea. Middle panel (C, D) red free images revealing similar underlying flecked retina. Lower panels (E, F) fundus autofluorescence showing marked hyper fluorescence associated with the yellow flecks scattered throughout the retina. Foveal region is spared but an arcuate region of hypofluorescence is visible superior to the foveae representing loss of the retinal pigment epithelium.

**FIGURE 2:**
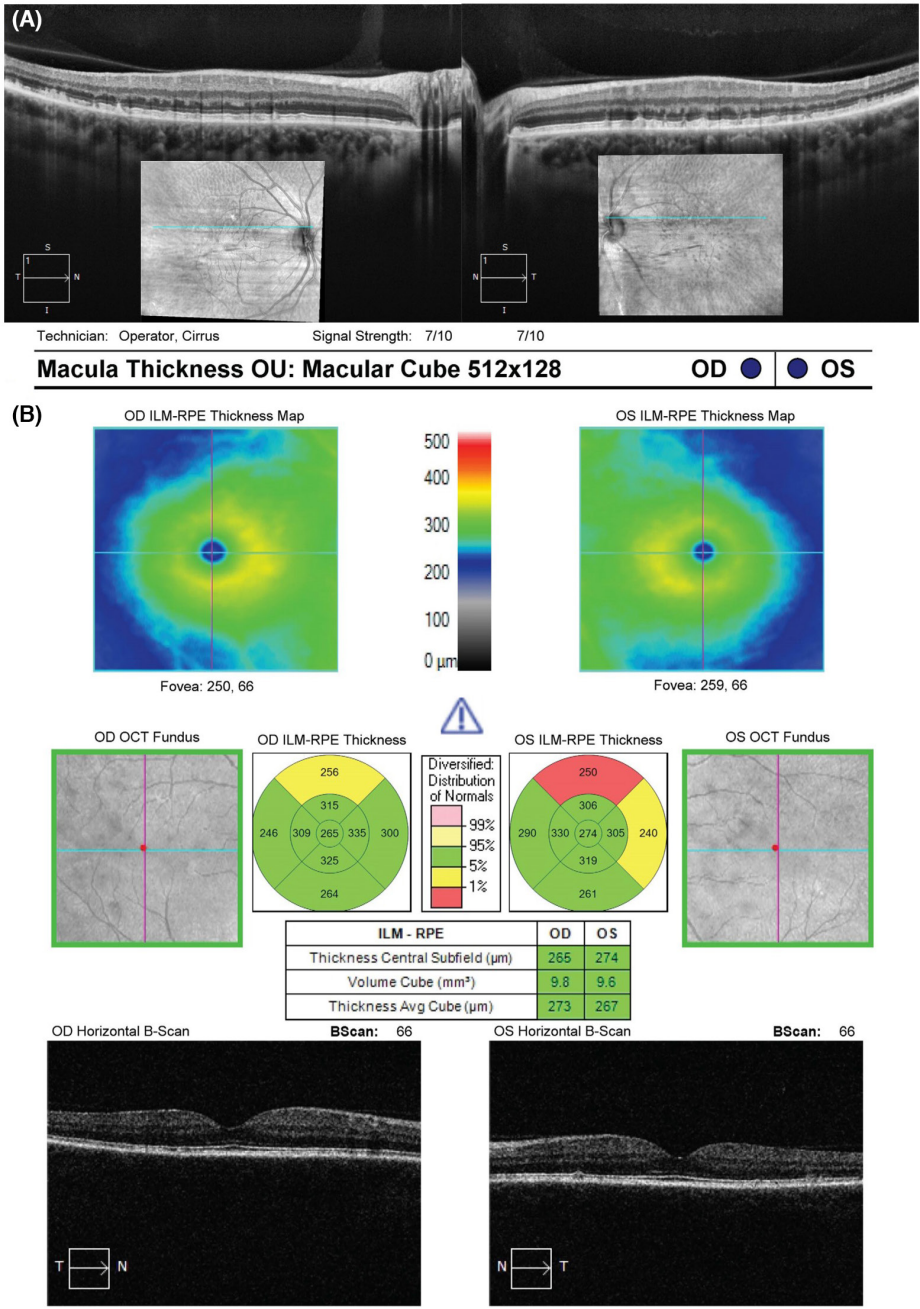
Upper images (A) shows OCT scan through the regions of retinal pigment epithelial atrophy with lesions present at the level of the RPE with overlying disruption to the ellipsoid zone in the right and left eye. Lower images (B) show thinning of the superior macula in the superior region with left eye greater than right and preservation of the foveae.

Fundus photos and red free revealed multiple yellow flecks in the peripheral retina with more punctate type lesions within the arcades. Fundus autofluorescence showed marked hyperfluorescence associated with the lesions as well as bilateral superior arcuate areas of hypofluorescence associated with retinal thinning. OCT revealed lesions at the level of the RPE‐Bruch's membrane and disruption to the ellipsoid zone in the presence of the retinal lesions. See Figures 1 and 2 for retinal imaging. The sister of the case had a normal fundus appearance with images in the Data S1.

### 2.1 Investigations

Full field dilated standard ISCEV ERGs were recorded using the RETeval (LKC Technologies, Gaithersburg, MD) with skin electrodes following either 20 min or 60 min dark adaptation (DA).^15^ All values are reported as the response value with the centile in brackets based on normative data from LKC Technologies. Following 20 min DA the DA0.01 b‐wave amplitudes were RE 26.6 μV (95th) and LE 24.3 μV (97th) with normal timings. Following 60 min DA the b‐wave amplitudes returned to within normal limits with RE 37.4 μV (21st) and LE 39.2 μV (26th). The DA3 a‐wave amplitude was outside normal limits for the RE −21.0 mV (98th) and LE −14.4 mv (100th) with within normal limits a‐wave timing and b‐wave amplitude and timing. The a‐wave amplitude did not recover following 60 min of dark adaptation with RE −23.4 μV (100th) and LE −24.7 μV (100th). Similar findings were observed with the DA10 with a reduced a‐wave amplitudes RE (−23.4 μV) and delayed time to trough 14.6 ms (100th) and LE a reduced a‐wave amplitude only −24.7 μV (100th). Following 60 min of dark adaptation the DA10 a‐wave amplitude recovered mildly but was still outside normal limits with RE −33.1 μV (97th) and LE −31.5 μV (98th) with normal a‐wave time to troughs OU and b‐wave amplitudes and time to peak.

**FIGURE 3:**
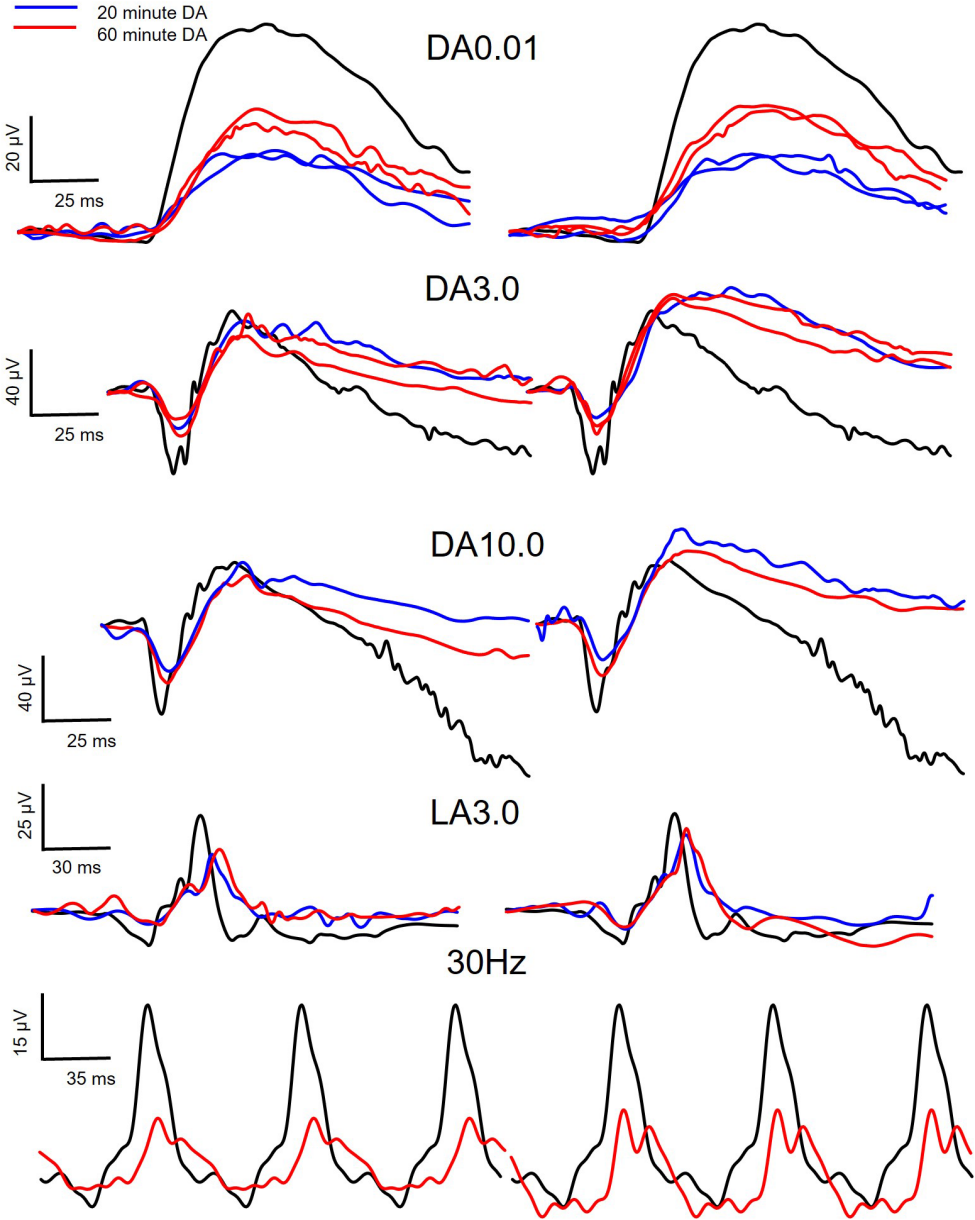
Electroretinograms after 20 min (blue) and 60 min (red) dark adaptation compared to a representative control (black). The DA0.01 b‐wave amplitude returned to normal levels after 60 min of dark adaptation. The a‐wave amplitude of the DA3.0 and DA10.0 failed was below normal limits after 20‐ and 60‐min dark adaptation. The LA3.0 responses were within normal limits although the right eye amplitude for the 30 Hz flicker was lower than normal. Right eye waveforms on the right‐hand side. For full reports see Data S1. DA, dark adaption.

Light adapted (LA) responses were overall within normal limits with values following 20 min dark adaption for the LA3 in the RE a‐wave amplitude −3.4 μV (11th) and −6.4 μV (46th) with normal time to trough and b‐wave amplitudes and time to peak. Following 60 min dark adaption the LA3 responses were similar with the a‐wave amplitude RE being −5.1 μV (30th) and LE −6.1 μV (43rd) with other parameters within normal limits. The 30 Hz flicker was performed after the 60‐min dark adaption period and the RE showed a significantly reduced amplitude 12.9 mV (98th) and normal LE amplitude 19.6 μV (19th) suggestive of some loss of function in the cones in the RE more than LE at this stage. See Figure 3 and Data S1 for full ERG reported waveforms.

### 2.2 Genetic screening

Next generation sequencing was performed on whole blood using whole exome capture (IDTxGen Exome v2) sequenced on the Illumina NextSEq sequencing system. Genes analyzed using the PanelApp Australia Retinal Disorders Superpanel v6.44 (Green and Amber). One homozygous variant of unknown significance was identified in *PLA2G5* c.40 + 5del containing a single nucleotide substation in intron 2 of 4 at +5 nucleotides from the last nucleotide of exon 2. This variant (rs1364254561) has not been previously described and is absent from the population database (PM2). Computational analysis supports a damaging effect, predicting the loss of the normal wildtype splice donor site (Alamut Visual Plus 1.6.1, SpliceAI) (PP3).

## 3 DISCUSSION

This case highlights an atypical presentation of FBFR with a novel variant in *PLA2G5* resulting in a loss of retinal function. FBFR is part of a group of retinal conditions that present with yellow to white flecks in the retina that spare the macula.^6^, ^7^, ^8^, ^9^, ^10^, ^11^, ^12^, ^13^, ^14^ Given the partial recovery of rod function with extended dark adaption in this case and adult presentation of symptoms our initial suspicions were directed to fundus albipunctatus and an *RDH5* mutation.^17^ When compared to previous cases of FBFR, this case has common features such as normal color vision, visual acuity, and a familial history of consanguinity; however, the abnormal ERG findings are not typically part of the clinical picture with usually normal retinal function. Given the normal ocular findings in her family there is evidence supporting an autosomal recessive pattern of inheritance consistent with FBFR.

In the most detailed description of the genetic variants associated with FBFR Sergouniotis et al.^6^ describe pathologic changes in one family consisting of a homozygous missense change c.133G > T (p.Gly45Cys) in exon 3 of *PLA2G5* and homozygous nonsense mutation c.185G > A (p.Trp62X), altering the last base of exon 3 in a separate individual. Two heterozygous changes c.133G > T (p.Gly45Cys) and c.383delA (p.Gln128ArgfsX88) were also identified in an unrelated individual, with no pathological mutation found in one further unrelated individual. The authors confirmed localization of phospholipase A2 with immunohistochemistry to be predominantly expressed in the inner and outer plexiform layers in an elderly human donor retina, in contrast to the main deposition of lipofuscin at the level of the retinal pigment epithelium. The authors speculate that this may be due to the role of phospholipase A2 reducing phagosome maturation,^3^ thereby reducing the ability of the retinal pigment epithelium to phagocytose outer segments.

Genetic screening identified a homozygous variant in intron 2 of *PLA2G5* c.40 + 5del may produce a splice variant of phospholipase 2 and result in the characteristic flecked retina with lipofuscin deposits sparing the macula. The current American College of Medical Genetics (ACMG) classification classes this as a variant of uncertain significance. Phenotypically, the electrophysiological findings are more akin to fundus albipunctatus with a delayed recovery of the dark‐adapted ERGs that characterize a loss of function in an enzyme such as 11‐cis‐retinol dehydrogenase encode by *RDH5* as a likely candidate.^16^, ^17^, ^18^

Thus, this case is the first to report a novel mutation in an intronic region of *PLA2G5* with clinical features of both fundus albipunctatus and FBFR. Other potential differential diagnoses may be tamoxifen retinopathy that may present with small and discrete white yellowish refractile elements in the retina.^19^

The reduced a‐wave amplitudes under DA conditions suggest a loss of rod function and the reduced 30 Hz flicker amplitude in the right eye suggest early cone involvement in this case. Further genetic analysis on siblings and parents were not possible, but there is no reported family history of eye disease or difficulties with night vision. Further studies may help identify if the mutation identified has a direct pathological role in the clinical findings and the progression of the disease. Currently the case is managing with her daily routines and is aware of her reduced time to adapt to dark conditions. Clinicians should be alert to late onset retinal dystrophies and the need for clinical electrophysiology and genetic screening to identify possible pathogenic variants. Whilst the ISCEV standard series of ERGs may suffice in most cases it is important to consider extended protocols such as prolonged DA, extended flash to dissect the ON‐ and OFF‐ pathways^20^ or the x‐wave that is a DA cone response^21^ to aid diagnosis of retinal disorders. Inherited retinal dystrophies typically affect vision in early childhood, however, this case highlights a late onset retinal dystrophy presenting in midlife. Such dystrophies that affect the macular may be misinterpreted as aging changes and patients presenting in adulthood with signs or symptoms of a retinal dystrophy should be investigated using visual electrophysiology.^15^

## AUTHOR CONTRIBUTIONS

**Paul A. Constable:** Investigation; project administration; writing – original draft. **Lynne Loh:** Investigation; writing – review and editing. **John R. Grigg:** Conceptualization; methodology; writing – review and editing.

## FUNDING INFORMATION

This work was unfunded.

## CONFLICT OF INTEREST STATEMENT

The authors declare that they have no conflicts of interest.

## ETHICS STATEMENT

All procedures performed in this case report involving a human participant were in accordance with the ethical standards of Flinders University and with the Declaration of Helsinki and its later amendments. This report does not contain any studies with animals performed by any of the authors.

## CONSENT

Written informed consent was obtained from the patient to publish this report in accordance with the journal's patient consent policy.

## Supporting information


Data S1:

## Data Availability

Raw electroretinogram data available on request to Paul A. Constable.
