# Supplementary material for: Suspected case of benign familial fleck retina with functional loss
Source: Clin Case Rep. 2023 Dec 21;11(12):e8362. doi: 10.1002/ccr3.8362 (PMC10733792; doi:10.1002/ccr3.8362)
Supplement: Supplementary file 1 — Data S1: [file CCR3-11-e8362-s001.docx]

Supplementary Material

**Suspected case of benign familial fleck retina with functional loss**

Paul A Constable, Lynne Loh, John R Grigg

Contents

[Additional Information 2](#_Toc145678985)

[Sequencing 2](#_Toc145678986)

[Family History 2](#_Toc145678987)

[OCT imaging 2](#_Toc145678988)

[Supplementary tests 4](#_Toc145678989)

[Electrophysiology 5](#_Toc145678990)

[20 minutes Dark adaptation 6](#_Toc145678991)

[60 minutes dark adaptation 7](#_Toc145678992)

[8](#_Toc145678993)

[9](#_Toc145678994)

# Additional Information

# Sequencing

Next generation sequencing was performed on whole blood using whole exome capture (IDTxGen Exome v2) sequenced on the Illumina NextSEq sequencing system. Genes analysed using the PanelApp Australia Retinal Disorders Superpanel v6.44 (Green and Amber). One homozygous variant of unknown significance was identified in *PLA2G5* c.40+5del containing a single nucleotide substation in intron 2 of 4 at +5 nucleotides from the last nucleotide of exon 2. This variant (rs1364254561) has not been previously described and is absent from the population database (PM2). Computational analysis supports a damaging effect, predicting the loss of the normal wildtype splice donor site (Alamut Visual Plus 1.6.1, SpliceAI) (PP3).

# Family History

The case’s family history indicated a history in the grand parents of consanguinity between cousins owing to their remote and isolated village in the Italian Alps. No direct relations (siblings or children) reported any symptoms or had retinal signs of a retinal dystrophy. See Figure 1 for currently known family pedigree.


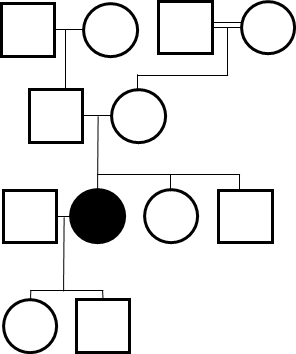


**Figure 1.** Family pedigree of the affected case (filled circle) with a history of consanguinity in the grandparents between cousins.

# OCT imaging

Retinal OCT scans revealed disruption to the retinal pigment epithelium and outer retina with hyperreflective deposits. Both superior sectors of the macula had thinning consistent with RPE atrophy (left > right) in this area.

**
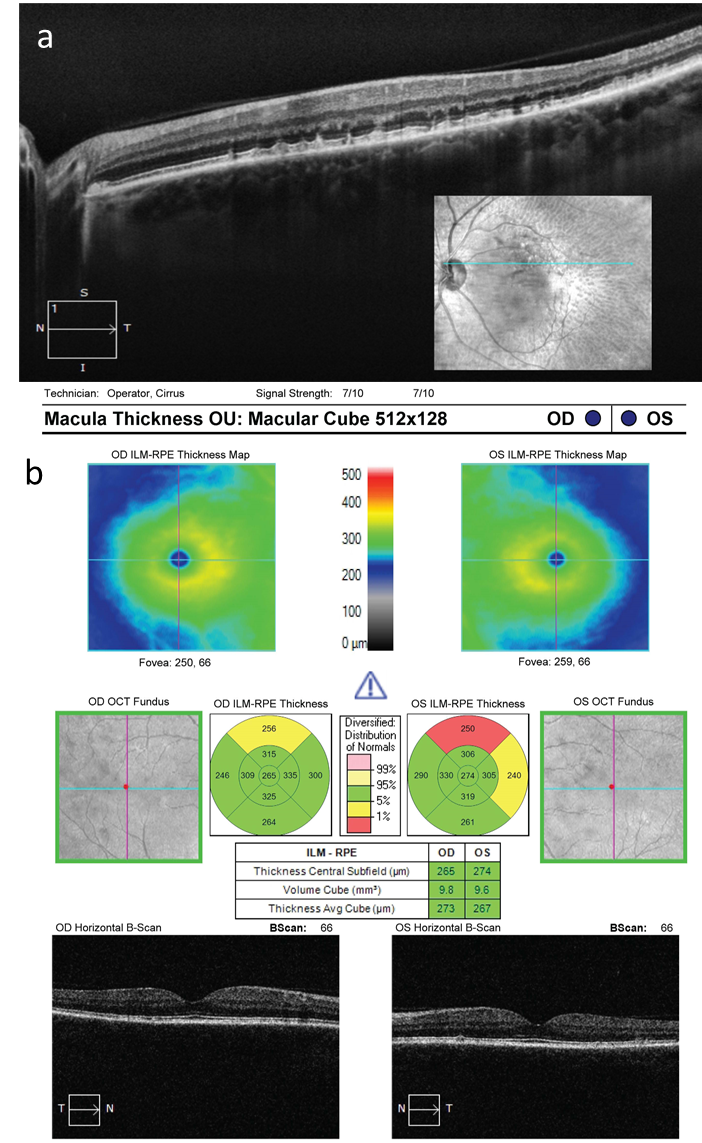
**

**Figure 2.** Upper images (a) shows OCT scan through the regions of retinal pigment epithelial atrophy with lesions present at the level of the RPE with overlying disruption to the ellipsoid zone in the right and left eye. Lower images (b) show thinning of the superior macula in the superior region with left eye greater than right and preservation of the foveae.

Figure 3 shows the normal fundus appearance of the sister of the case. The case reported no family history of nyctalopia in her siblings (sister and brother) parents and two children (male and female) supporting an autosomal recessive inheritance pattern.


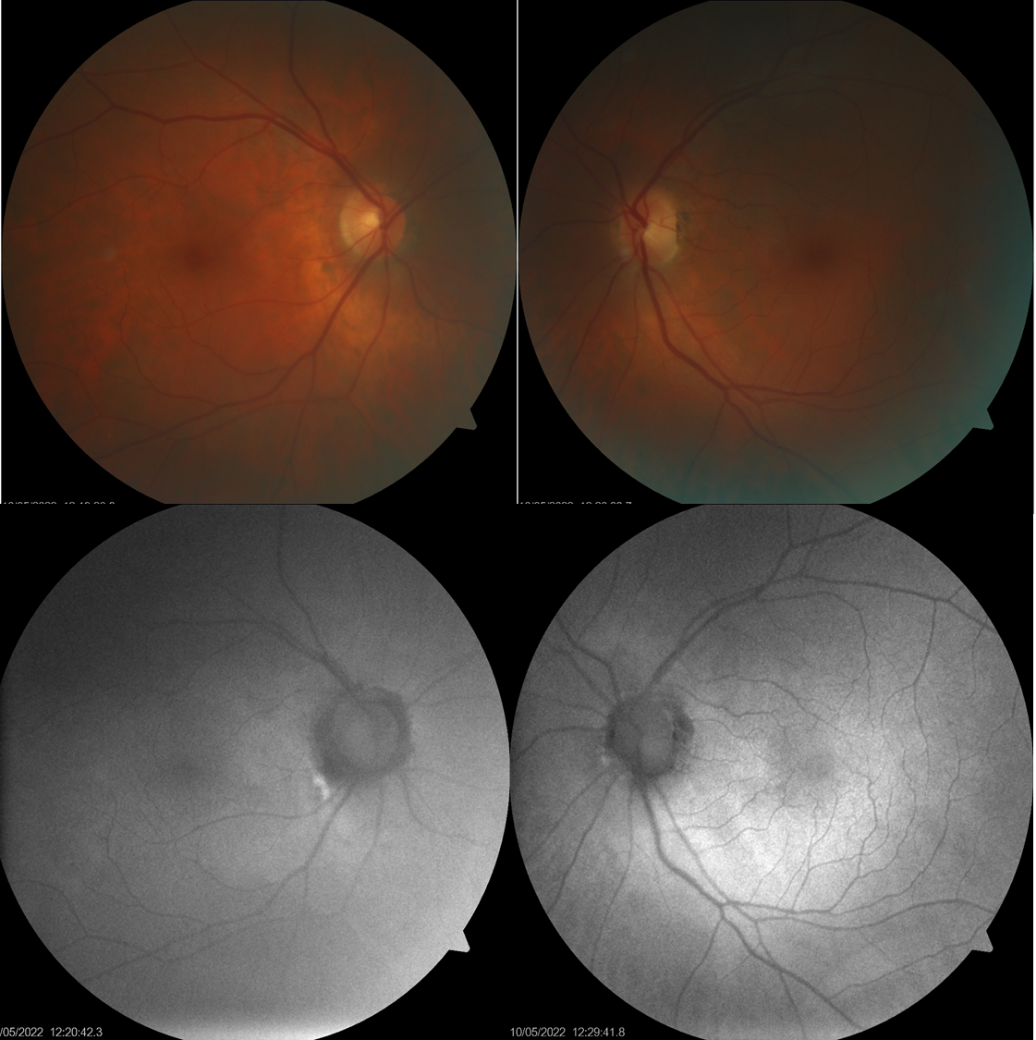


**Figure 3.** Upper panel shows colour fundus images with lower panel red-free images of the sister of the case. No abnormalities were apparent.

# Supplementary tests

At presentation, in March 2022, the case’s refractive error was (RE +0.50/-0.25x177 and LE +0.75/-0.50x87 Add +2.25) with visual acuities 6/6 OU and IOPs of 13 mmHg OU (Goldmann applanation). Visual fields HFA-II 24-2 were normal with mean deviation RE -0.03dB and LE -0.90 dB). Colour vision (binocular) with the D-15 was normal. See Figure 3 for Humphrey central 30-2 visual fields which together with normal colour vision supporting normal photopic vision.


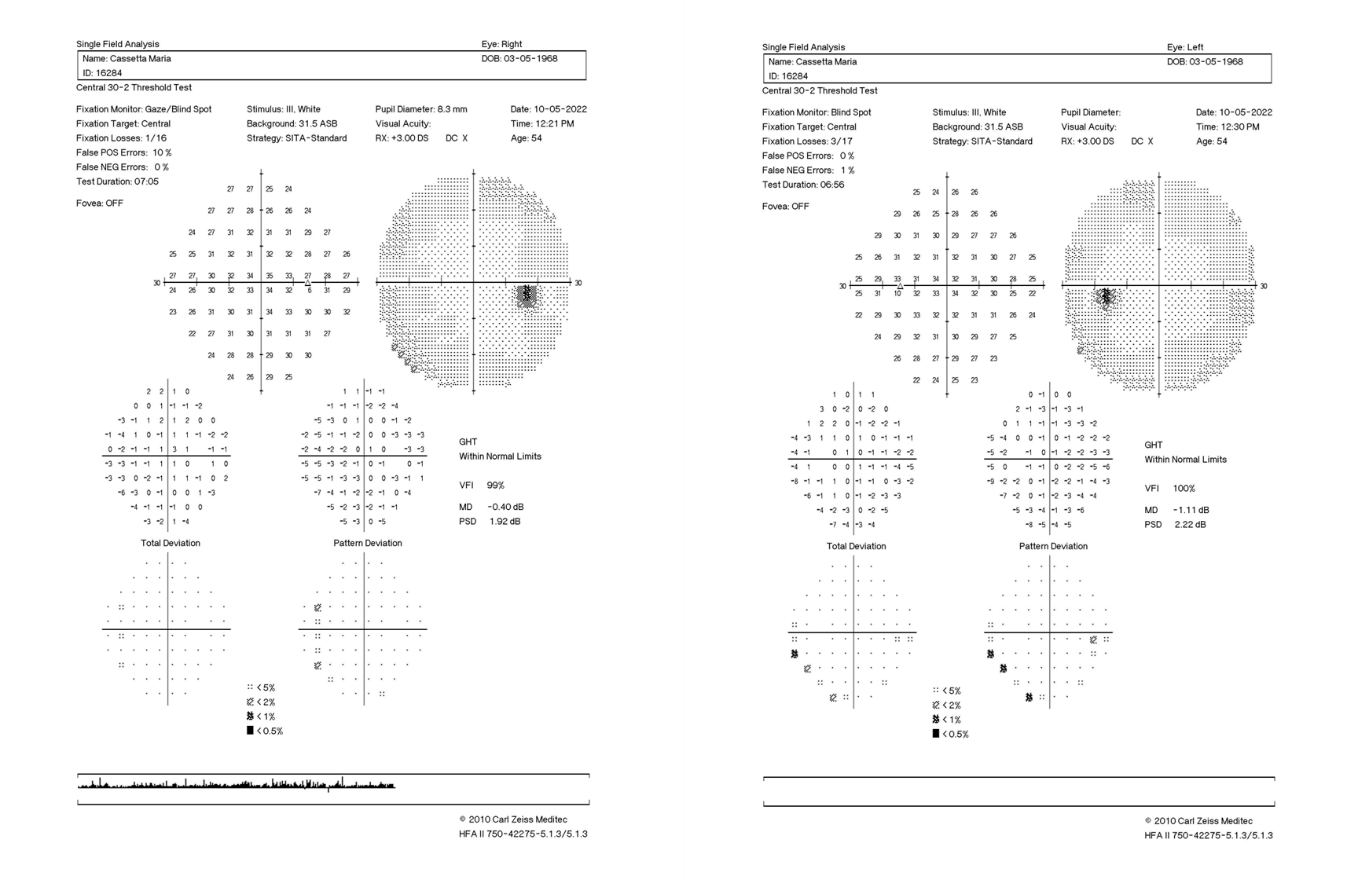


**Figure 4.** Humphrey visual field plots of the case at presentation with no marked central or peripheral scotoma.

# Electrophysiology

Full field dark and light adapted (DA and LA) ISCEV standard electroretinograms were recorded from the right then left eyes after 20- and 60-minutes dark adaptation to evaluate recovery of the rod driven retinal pathways. The subject was dilated with 1% Tropicamide, and recordings were performed with skin electrodes and the RETeval 6 step Troland ISCEV protocol. Raw traces are shown below of the recordings for the DA 0.01, DA3, DA10, LA3 and 30 Hz flicker.

Amplitudes and time to peak of the a- and b-waves are indicated with values falling outside the lowest 5^th^ centile for the normal reference range indicated by yellow or red highlights. Infra-red image sof the subject’s eye and electrode are shown in the report outputs.

## 20 minutes Dark adaptation


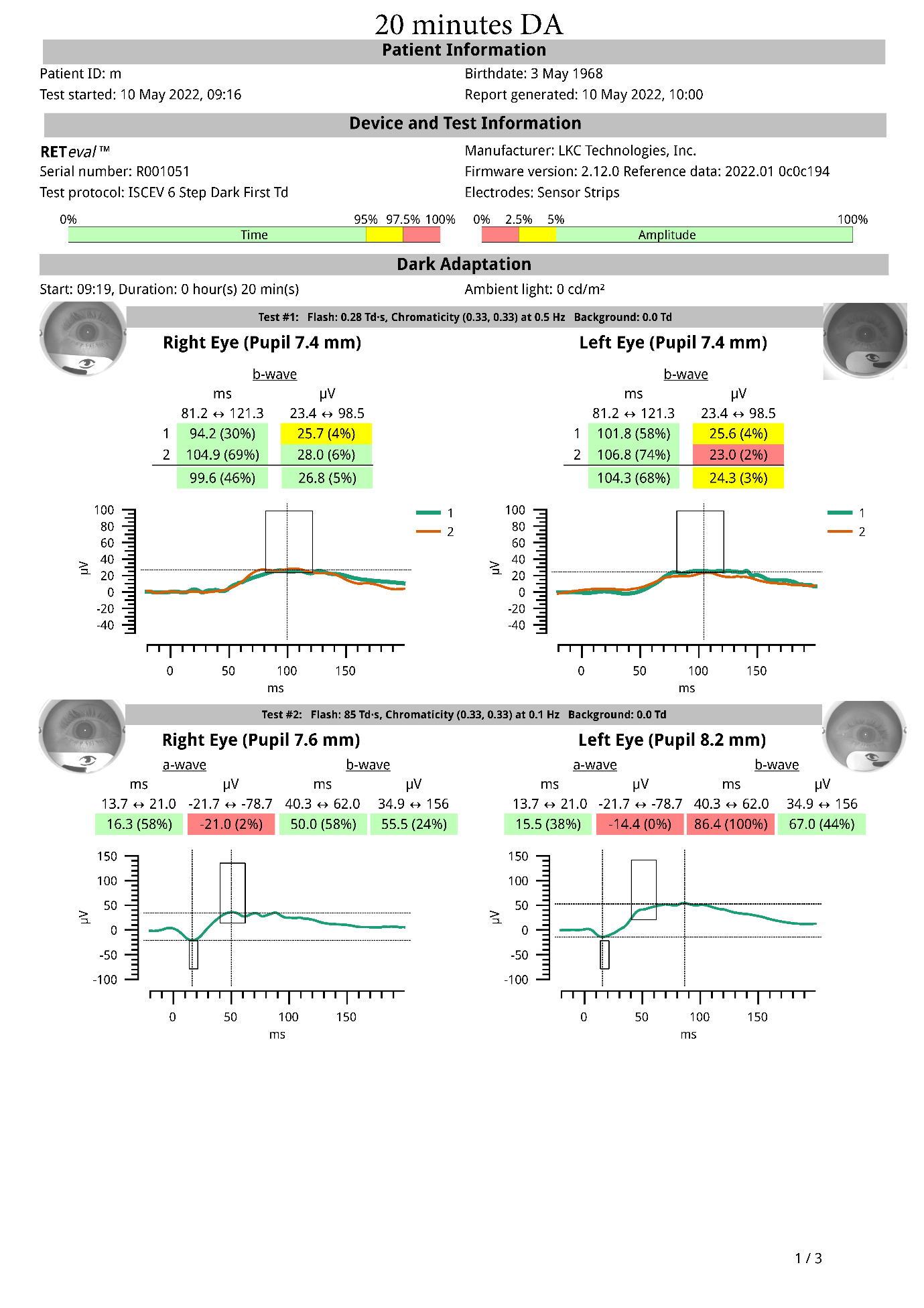


Twenty minutes Dark adaptation- DA0.01 upper panel at 20 minutes DA with borderline reduction in the rod driven b-wave amplitude consistent with a loss of rod function. Lower panel the DA3 with reduced a-wave amplitude suggesting reduced rod phototransduction with decreased hyperpolarisation of the rod outer segments.


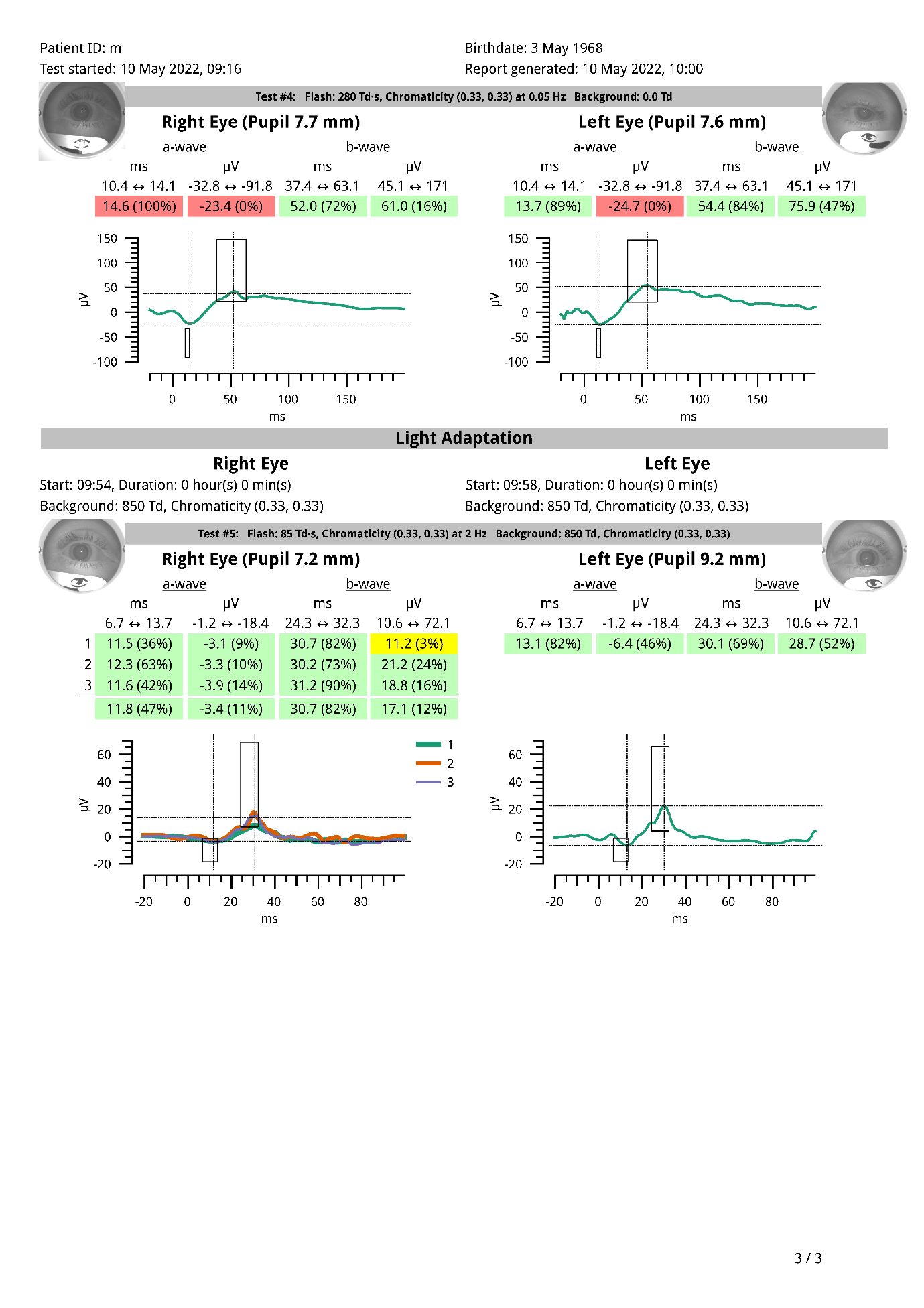


Twenty minutes dark adaptation – upper panel showing the brighter DA10 with significant loss of the a-wave amplitude supporting a loss of rod function. Lower panel show the light adapted LA3 cone driven responses which are normal.

## 60 minutes dark adaptation

The ERG recordings were repeated after 60 minutes of dark adaptation to evaluate recovery of the rod pathways.

##
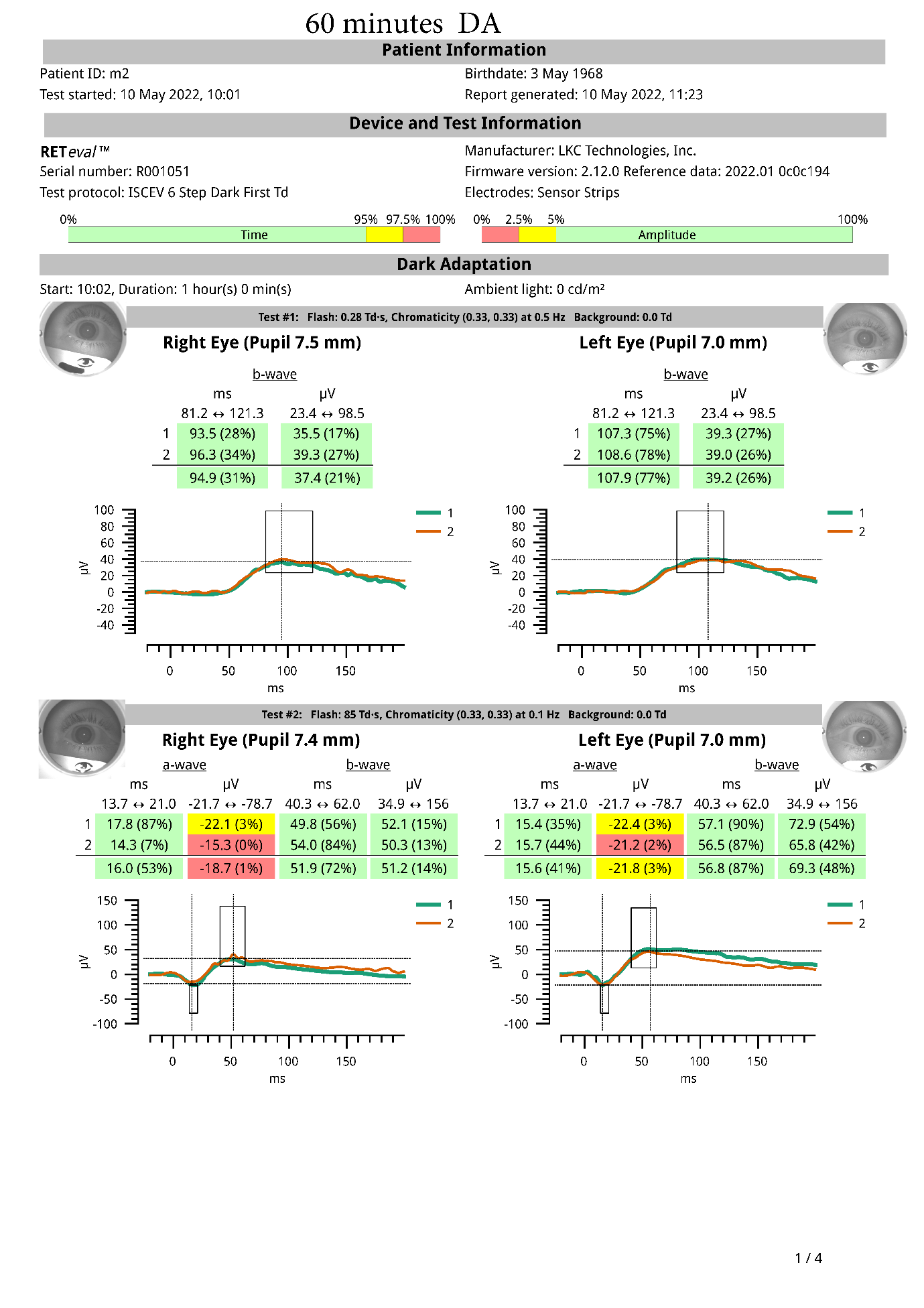


Following 60 minutes of dark adaptation the DA0.01 b-wave recovered to within normal limits whilst the a-wave of the DA3 improved but was still outside normal limits.

##
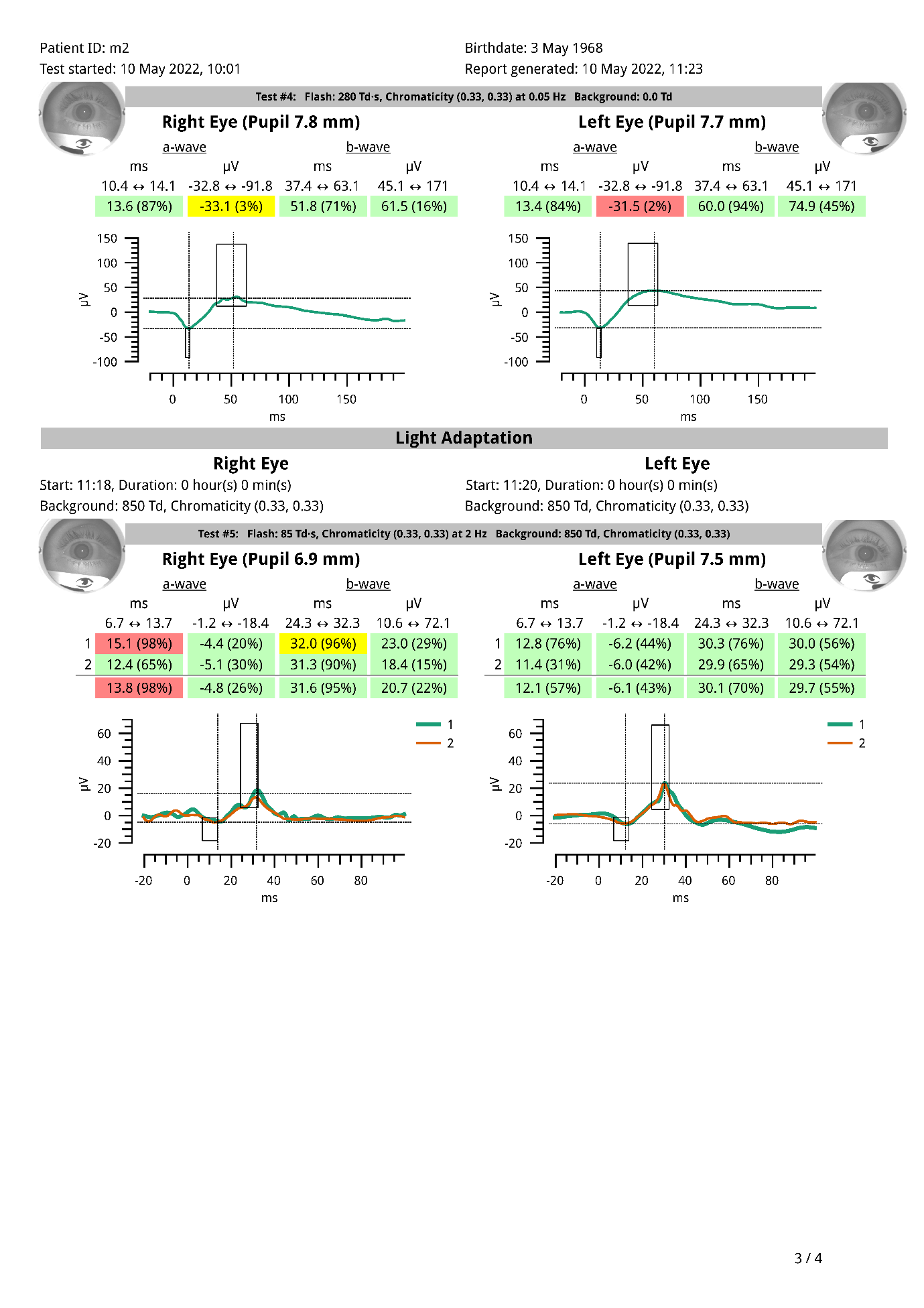

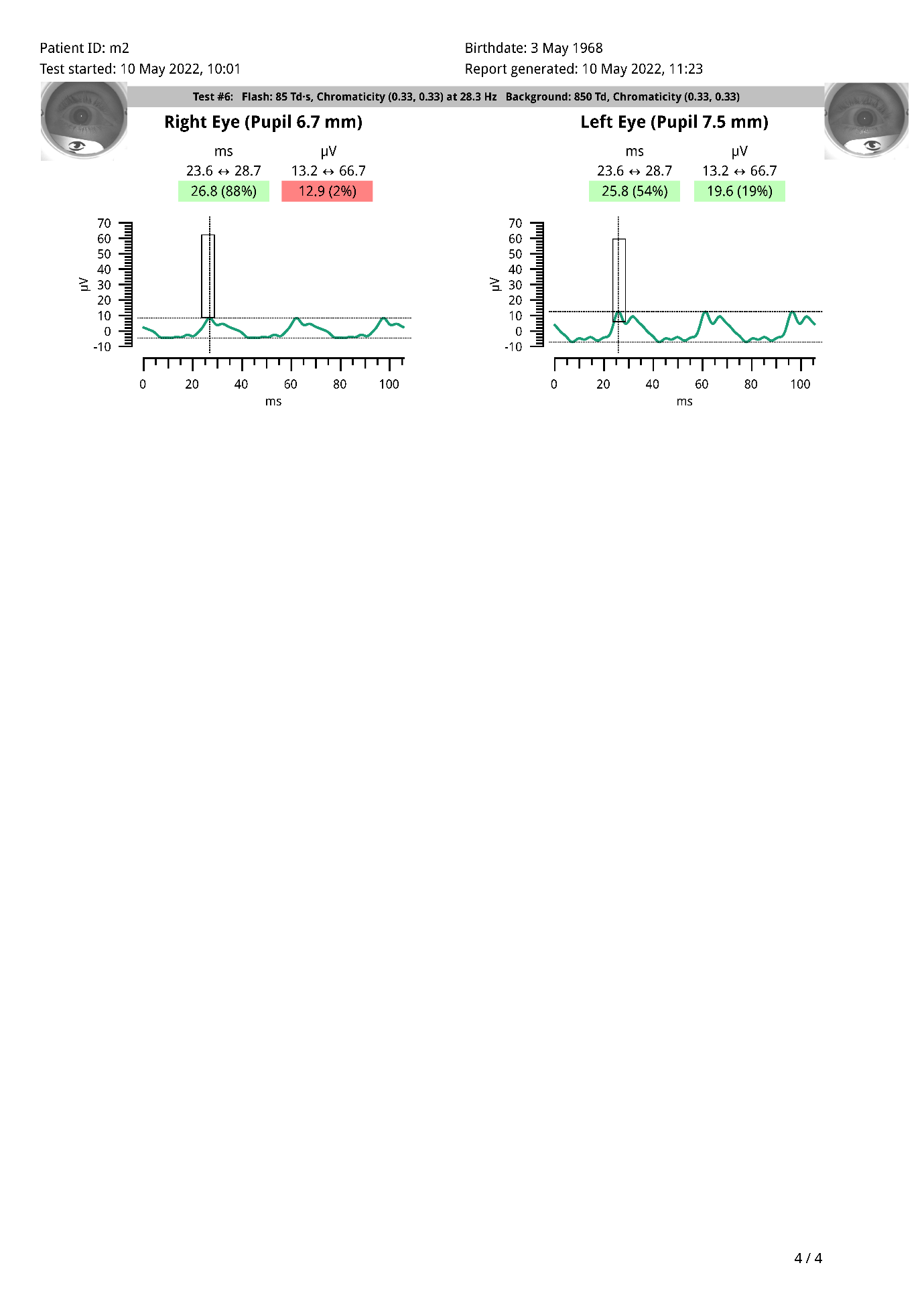


60 minutes dark adaptation for the DA10 showed impairment in the a-wave amplitude with normal LA3 cone responses. There was a slight reduction in the 30Hz flicker amplitude in the right eye suggesting some involvement of the cones in the right eye.
